# Improved Support Vector Machine Enabled Radial Basis Function and Linear Variants for Remote Sensing Image Classification

**DOI:** 10.3390/s21134431

**Published:** 2021-06-28

**Authors:** Abdul Razaque, Mohamed Ben Haj Frej, Muder Almi’ani, Munif Alotaibi, Bandar Alotaibi

**Affiliations:** 1 Department of Computer Engineering and Information Security, International Information Technology University, Almaty 050040, Kazakhstan; 2 Department of Computer Science and Engineering, University of Bridgeport, Bridgeport, CT 06604, USA; mbenhaj@bridgeort.edu; 3 Gulf University for Science and Technology, Hawally 32093, Kuwait; almiani.m@gust.edu.kw; 4 Department of Computer Science, Shaqra University, Shaqra 15526, Saudi Arabia; munif@su.edu.sa; 5 Department of Information Technology, University of Tabuk, Tabuk 47731, Saudi Arabia; b-alotaibi@ut.edu.sa; 6 Sensor Networks and Cellular Systems (SNCS) Research Center, University of Tabuk, Tabuk 47731, Saudi Arabia

**Keywords:** remote sensing, support vector machine, improved SVM-RBF variant, improved SVM-Linear variant, image classification

## Abstract

Remote sensing technologies have been widely used in the contexts of land cover and land use. The image classification algorithms used in remote sensing are of paramount importance since the reliability of the result from remote sensing depends heavily on the classification accuracy. Parametric classifiers based on traditional statistics have successfully been used in remote sensing classification, but the accuracy is greatly impacted and rather constrained by the statistical distribution of the sensing data. To eliminate those constraints, new variants of support vector machine (SVM) are introduced. In this paper, we propose and implement land use classification based on improved SVM-enabled radial basis function (RBF) and SVM-Linear for image sensing. The proposed variants are applied for the cross-validation to determine how the optimization of parameters can affect the accuracy. The accuracy assessment includes both training and test sets, addressing the problems of overfitting and underfitting. Furthermore, it is not trivial to determine the generalization problem merely based on a training dataset. Thus, the improved SVM-RBF and SVM-Linear also demonstrate the outstanding generalization performance. The proposed SVM-RBF and SVM-Linear variants have been compared with the traditional algorithms (Maximum Likelihood Classifier (MLC) and Minimum Distance Classifier (MDC)), which are highly compatible with remote sensing images. Furthermore, the MLC and MDC are mathematically modeled and characterized with new features. Also, we compared the proposed improved SVM-RBF and SVM-Linear with the current state-of-the-art algorithms. Based on the results, it is confirmed that proposed variants have higher overall accuracy, reliability, and fault-tolerance than traditional as well as latest state-of-the-art algorithms.

## 1. Introduction

As a vital tool for information retrieval regarding land cover and land use, remote sensing (RS) technologies have widely been used in various areas (e.g., land management, and urban and rural planning) [1,2,3]. RS is the method that provides information about events by assessing the data. The data is collected using special instruments, which do not interact physically with the environment under study [4]. Thus, the Knowledge of land-cover/land-use is vital in a number of arenas based on the observations done for the metropolitan and regional future planning [5]. In the area of sustainable development, image classification in RS can be used to assess changes in different ecosystems—namely, to monitor global climate change, to assess natural disasters, to track forest fires, to determine air pollution and to observe air quality [6]. Compared with field investigation, RS technology is much more efficient and cheaper in terms of time and cost [7]. RS image classification is a significant part of the overall field of RS, which can be thought of as a joint venture between both image processing and classification techniques [8]. The classification can be implemented by algorithms that are either supervised or unsupervised: the former uses pre-labeled data and the latter uses data without labeling. In RS classification, supervised classification algorithms are usually preferred due to their accuracy and practicability [9,10,11]. The statistical distribution of data can severely decrease the accuracy when the data do not follow those assumptions.

MDC and MLC can produce different accuracies and efficiencies based on their mathematical principles. An analysis of MLC is elaborated by [12]. However, as parametric classifiers, the algorithms are severely affected by several assumptions. MDC requires the distribution of the mean vectors of each class to be much wider than the distribution of the training data within each class, while MLC assumes that the statistics for each training class obey a multivariate normal distribution. Several algorithms have been introduced to provide the great accessibility for high-quality image procurement [13]. The SVM was first developed to deal with binary classification, and the explanation above assumes that there are simply two classes to be classified. For most of the cases in RS imagery, there are more than two classes of land use [14,15]. Thus, some techniques need to be adopted to transfer the binary classifier to a multiple classifier. The SVM has a capability to train and test the feature vectors and works efficiently with unstructured and semi structured data [16]. As a result, it works fast with linearly separable. vectors and low-dimensional features. The SVM classifier has a disadvantage because it does not support several overlapping classes. It is also delicate with the noisy feature vectors. To improve the classification accuracy by removing the constraints in parametric classifiers, our proposed variants adopt the structural risk minimization principle, which does not assume the distribution of data, although parametric classification algorithms have a higher time complexity due to their complex mathematical principles, these algorithms usually have higher accuracy and are more stable when dealing with small samples. In this paper, we further assess the performance of current state-of-the-art algorithms and compared them to our proposed SVM-RBF, and SVM-Linear. The main contribution of the paper is summarized as follow:

- Novel framework based on SVM-RBF and SVM-Linear for the classification of remote sensing images have been introduced to improve the accuracy and efficiency and overcome many existing challenges.
- The proposed SVM-RBF and SVM-Linear are capable to address mask generation, cross-validation, ranking, change classification/No-change classification, underfitting, and overfitting.
- SVM-Linear and non-linear SVM-RBF can minimize the computational load by separating the samples from different classes.
- The SVM-RBF and SVM-Linear are also compared with the state-of-the-art algorithms (NDCI, SCMask R-CNN, CIAs, KCA, and AOPC from the change detection accuracy, and reliability perspective. The proposed SVM-RBF and SVM-Linear have shown higher overall accuracy and better reliability compared to existing approaches.

The remainder of the paper is organized as follows. Section 2 presents salient features of the existing approaches. Section 3 presents materials and methods. Section 4 discusses and gives an overview of the mathematical principles. Section 5 presents experimental results, and finally the entire paper is concluded in Section 6.

## 2. Literature Review

In this section, the salient features of the existing approaches are summarized. The analysis of supervised classification algorithms in image classification has been a trending topic among information technology specialists, as improving classification accuracy is vital for RS to have a practical use. Some researchers have focused for the most part on the evaluation of parametric classifiers. Improved Mask of Recurrent Neural Network is introduced remote sensing images [17]. The proposed approach is called as SCMask R-CNN, and the goal of this proposed approach is to enhance the detection effect by providing a higher resolution of remote image sensing.The proposed approach also provided segmentation and object recognition concurrently. In [8], the authors have focused on the discoloration and modification in the optical processes. Thus, new non-destructive and content independent (NDCI) methods are proposed for ranking classification. The proposed approach is tested on multispectral images for determining the spectral responses. However, the proposed approach is not highly compatible with remote image sensing. The computational intelligence applications (CIAs) are evaluated in the remote image sensing process [18]. The authors in [18] address many challenges that face the applications of remote sensing images, such as high-dimensional data, complex data structures, and the nonlinear optimization issues.

The evaluation process focused on the feature demonstration and selection. The clustering, classification, and change-detection processes are performed. Consequently, the fundamental capacities of computational intelligence are described from the remote image sensing perspective. However, the evaluation failed to provide mask generation, ranking, underfitting, and Overfitting. Multi-temporal hyperspectral remote sensing (HSRS) is compatible with image change detection. Thus, the authors in [19] proposed an HSRS based on deep learning and tensor. The organization mode has been optimized to preserve the integrity between dissimilar underlying features. The large quantity of unlabeled and untagged samples are trained using multilayer Tensor3- Restricted Boltzmann Machine. Finally, the traditional Boltzmann Machine based on neural networks is replaced with a support tensor machine to obtain the land-use changes. The results demonstrate that the proposed has higher change detection accuracy as compared to other methods and also provides a better automation level. Although the accuracy assessments of supervised classification algorithms in RS have already been studied by many researchers, most of the assessments are only focused on the accuracy of specific datasets but neglect the poor manner with which the problem is posed [20]. High accuracy in one specific dataset can sometimes be deceptive because of overfitting. In that case, the algorithm cannot be applied to practical use well due to its low accuracy with new data. Consequently, the accuracy in the test set is also of great significance in accuracy assessment to address the problem of overfitting. Thus, ref. [21] studied the performance of the four algorithms (K-nearest neighbor (KNN), SVM, Random Forest (RF), and Artificial Neural Network (ANN)) to determine the desert-oasis medley landscapes merging Key Component analysis (KCA). Based on the results, the authors declared the RF algorithm as the first choice. However, they failed to justify the impact of RF on the overall accuracy.

The overfitting and poor generalization problems are discussed in [22]. The proposed Addressing Overfitting on Pointcloud Classification (AOPC) aims to address the inducing controlled noise generated by conditional random field parallel penalties using adjacent features of [22]. The authors proposed new algorithm named Atrous XCRF to overcome the overfitting problem and enhance the classification of pointcloud data. The proposed method is used for training and utilizing the unlabeled part of a dataset to improve model accuracy. The proposed method gets better accuracy, but it still suffers due to generalization problems. The SVM experiences the problem due to different sorts of vulnerabilities, such as authentication bypass, context escape, trust failure, and code execution [23]. The localization vulnerability is a big issue that leads to possible attacks on the memory, network traffic, and file system. As a result, these localization problems cause service interruption. The SVM is particularly designed to handle binary classification. Thus, multiclass classification can be the big issue for applying the SVM. Another problem encountered by the SVM is the extended training and testing time because it behaves poorly during the extended training and testing time. The SVM is not fully capable of attack detection because it takes more time to be trained and tested. The main goal of this research is to introduce robust SVM variants to yield lower false-positive rates and produce higher classification accuracy [24].

The objective of our research is to create a land-use classification method using new SVM-RBF, SVM-Linear, MLC and MDC, and other current state-of-the-art algorithms. The SVM-RBF, SVM-Linear, MLC, and MDC have been compared both from a mathematical perspective and with the experiment results to show their different levels of accuracy and efficiency. Moreover, SVM-RBF, SVM-Linear have also been compared with current state-of-the-art algorithms: NDCI [8], SCMask R-CNN [17], CIAs [18], KCA [21], and AOPC [22] from the change detection accuracy, and reliability viewpoint.

We believe that our proposed SVM-RBF and SVM-Linear are capable to address the mask generation, cross-validation, ranking. change classification/No-change classification, underfitting, and overfitting. We further argue that the thorough analysis and comparison will help others to design a better classification algorithm for the land cover and parameter settings. Table 1, demonstrates the features of algorithms for the land cover and land use properties.

The vulnerabilities and the security issues are not handled in this article. The adversarial examples can also add little noise to the original image that leads to misclassification. We will try to propose a similar type of restricted adversarial example solution for our proposed variants in the future as introduced in [4] for deep neural network (DNN).

**Figure 1:**
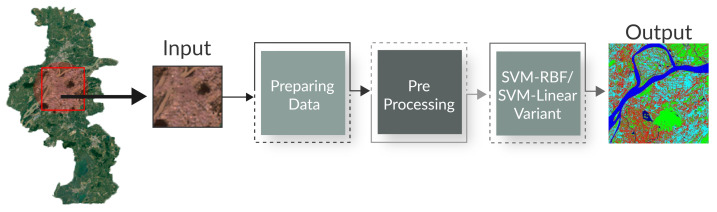
The proposed framework of the remote sensing image classification.

The general procedure of the proposed framework is as follow: reading the remote sensing image, pre-processing step for input image, and the classification step which is apply to each pixel in the image. Figure 1 shows the proposed framework for the classification of the remote sensing image.

## 3. Materials and Methods

### 3.1. Datasets

**Figure 2:**
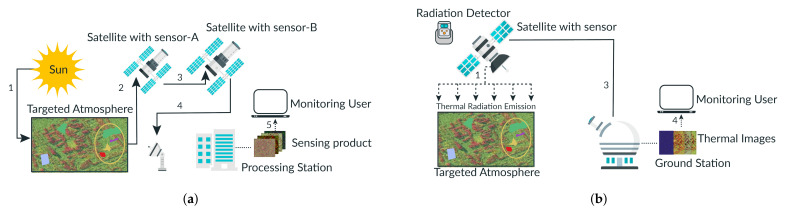
Passive remote sensing processes. (**a**) A passive remote sensing process using optical remote sensing; (**b**) A passive remote sensing process using thermal remote sensing.

The study area considered in this paper includes the regions of Xuzhou Jiangsu, China, and Tanintharyi, located in southern Myammar. In the Xuzhou Jiangsu, China, region, the coordinates of the area are 30${}^{\circ }$40″ to 31${}^{\circ }$40″ northern latitude and 117${}^{\circ }$40″ to 118${}^{\circ }$0″ eastern longitude, covering about 5000 km${}^{2}$. The radar satellite and optical data were combined and used for the land cover change evaluation. The Landsat-8 Operational Land Imager and Landsat-5 Thematic Mapper were used for optical data collection. Global L-band Synthetic Aperture Radar was used for radar data. Several images were taken using two types of RS methods (optical and thermal). Figure 2a,b show the schematic process of RS using optical and thermal. In both types of RS methods, a passive sensing system is applied that gets the energy from the sun to obtain electromagnetic energy. The electromagnetic interaction with the targeted atmosphere. In reply, the targeted atmosphere reflects, then satellite sensor-A records the emitted energy from the targeted atmosphere. The recorded energy is transmitted to satellite Sensor-B by the satellite Sensor-A. When a satellite sensor-A receives recorded energy, then it is transmitted to the processing station. Finally, the data is processed, evaluated, and construed to obtain the required image.

**Figure 3:**
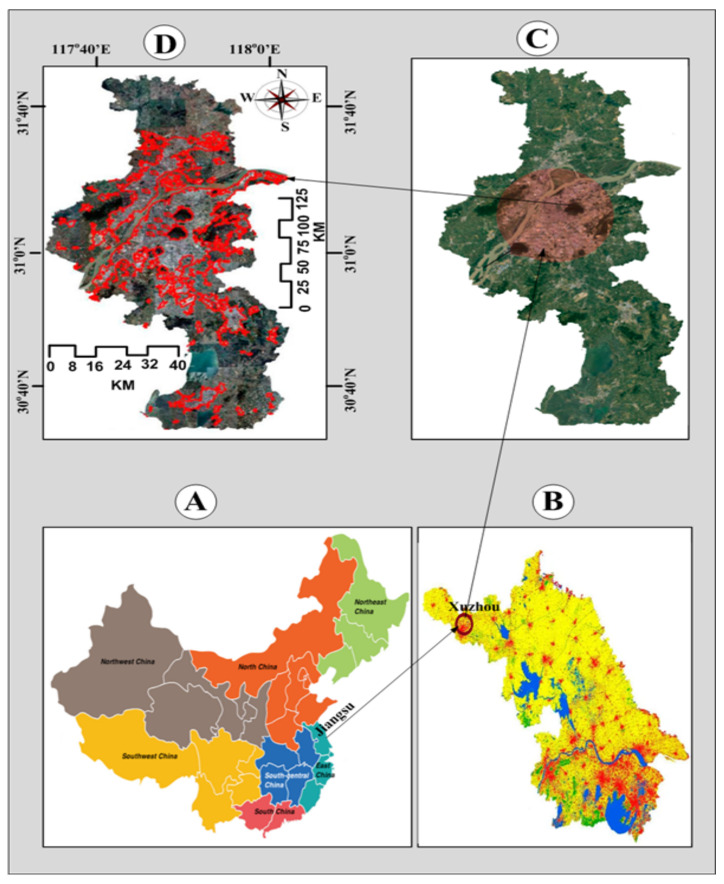
Remote sensing image of Xuzhou Jiangsu, China.

Images were also taken from human and non-human zones. One of the RS images covering different densities is depicted in Figure 3. The image shown in Figure 3 is taken from Landsat-8 TM [25].

**Figure 4:**
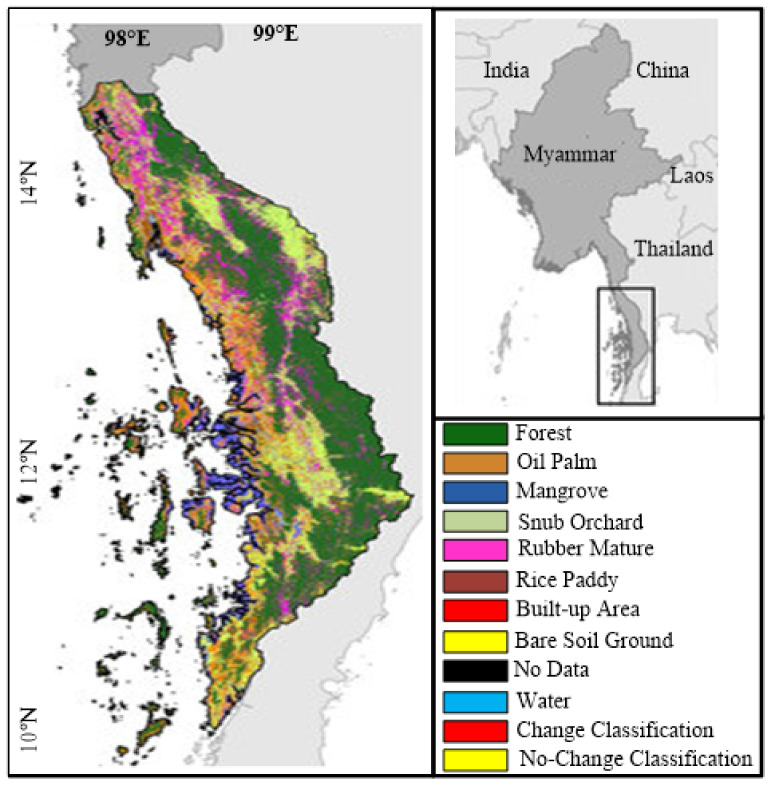
Image classification for the Tanintharyi region location in southern Myanmar.

As Figure 4 shows, 10 classes of the land are used in the reference map. To simplify our research, we chose change classification and no-change classification. Moreover, we implemented the image classification depicted in Figure 4 for the Tanintharyi region located in southern Myanmar, with coordinates of 9${}^{\circ }$ to 16${}^{\circ }$ northern latitude and 97${}^{\circ }$ to 100${}^{\circ }$ eastern longitude, covering about 43,345 km${}^{2}$. The coastal areas of the Andaman sea were focused to the west and the Tenasserim Hills to the east, with 2072 m being the highest elevation above sea level.

The land cover classes have been defined through a combination of the visual interpretation and field verification of the high-resolution images. The experiment was done with a reference map. The ground truth data were gathered from field verification from two different regions—Xuzhou Jiangsu, China and Tanintharyi, Myammar—in 2020. The sources used for collecting data were the Smithsonian Institution, Virginia, USA, EcoDev/ALARM, Myanmar and Institute of Remote Sensing and Digital Earth Chinese Academy of Sciences, China. The visual analysis of reference images was based on the features that help in the recognition of land cover geographies such as location, shape, size, color, shadow, tone, smoothness and shape. We used 7.6.4 (R.G.B) false-color synthesis, which is considered suitable for monitoring human and non-human zones.

### 3.2. Parameters in SVM-RBF and SVM-Linear Variants

The accuracy of the SVM is largely based on the choice of variants and parameters. For our study, we use the linear SVM-enabled-RBF and SVM-enabled-linear in order to show both linear and non-linear classification. The parameters in the RBF variant are the parameter *g* and penalty factor *C*. The penalty factor *C* is used for regularization, which deals with the problem of underfitting and overfitting in the cost function itself, while γ is unique to the RBF kernel.

**Figure 5:**
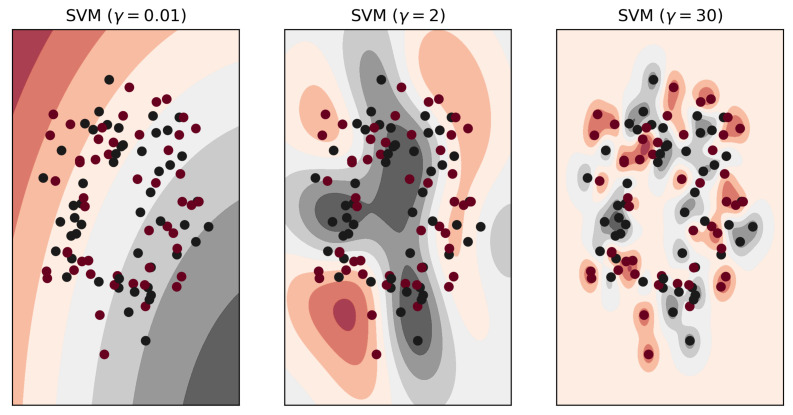
Kernel parameter γ for underfitting, regularized, and overfitting models respectively.

The kernel parameter Υ determines how many nearby samples the support vector will consider, which also addresses the problem of underfitting and overfitting indirectly. Figure 5 demonstrates the outcome of the underfitted, overfitted and regularized models.

In order to optimize those two parameters, we adopt a cross validation method that divides the training samples into two parts: the first part is used to train the model while the other is used as a test set to evaluate the accuracy using the current parameter. The cross validation is implemented using grid search of exponential growth parameters, with the default value being the median. The default values of the penalty factor *C* and kernel parameter γ are 100 and the reciprocal of the number of features, which is 0.33 in our case. In our case, *C* and γ are selected from set$\left\{2^{-4}\times 100...2^{-1}\times 100,\;100,\;2^{1}\times 100...2^{4}\times 100\right\}$ and $\left\{2^{-4}\times 0.33...2^{-1}\times 0.33,\;0.33,\;2^{1}\times 100...2^{4}\times 100\right\}$ respectively, constituting 9×9=81 permutations to test.

### 3.3. Image Processing

Before classification, some pre-processing techniques can be applied to calibrate the original image, such as radiometric, atmospheric and geometric corrections, removing the undesired impact of irrelevant data. This is not our focus, but image processing has an effect on the comparison of algorithms. The Landsat image composite is of paramount importance and requires five indexes, which can be calculated as follows:

(1) $$N_{vi}=\frac{\left(N_{p}-R_{p}\right)}{\left(N_{p}+R_{p}\right)}$$

(2) $$E_{vi}=2.5*\frac{\left(N_{p}-R_{p}\right)}{N_{p}+\left(6.0*R_{p}-7.5*B_{p}\right)+1.0}$$

(3) $$S_{vi}=\frac{\left(S_{ir1}-R_{p}\right)}{\left(S_{ir1}+R_{p}+0.1\right)}*\left(1.1-\frac{S_{ir2}}{2.0}\right)$$

(4) $$N_{ti}=\frac{\left(S_{ir1}-S_{ir2}\right)}{\left(S_{ir1}+S_{ir2}\right)}$$

(5) $$L_{sw}=\frac{\left(N_{p}-S_{ir1}\right)}{\left(N_{p}+S_{ir1}\right)}$$

where $N_{vi}$ is the normalized difference vegetation index; $N_{p}$ is the near infrared band; $R_{p}$ is a red band; $B_{p}$ is a blue band; $E_{vi}$ is the enhanced vegetation index; $S_{vi}$ is the soil-adjusted entire vegetation index; $S_{ir1}$ is the shortwave infrared index-1; $S_{ir2}$ is the shortwave infrared index-2; $N_{ti}$ is the normalized difference tillage index; and $L_{sw}$ is the land surface water index. We applied optical indexes due to nature of land cover and forest. $N_{vi}$ and $L_{sw}$ are used to gain better separation between the land cover types of forest and croplands. $N_{ti}$, $S_{vi}$ and $L_{sw}$ are leading predicators for distinguishing between forests and plantations. $E_{vi}$, $N_{vi}$ and $L_{sw}$ are useful for forest-mapping and broad-leaved plantations [26].

### 3.4. Selection of Training Test and Testing Set

In RS, regions of interest (ROIs) are chosen manually, which work in a similar manner to training data. During the training procedure, the ROIs specify the region in which the objects are chosen that the computer should learn [27]. The image statistics are extracted to generate the masks. The box-whisker plots are constructed from the image statistics to visualize reflectance-distribution values for each covered land type against each radar/optical channel to determine the predicator variables. The predicator variables are capable of discriminating the land cover classes, which are picked based on a visual evaluation of the plots. The classes are separable based on dissemination.

Training areas were established by choosing one or more polygons for each class [28]. After choosing ROIs, we implemented a random shuffle algorithm for ROIs by modifying the interactive data language (IDL) in an environment for visualizing images (ENVI) and chose the first 90% as the training data set and the remaining 10% as the test data set.

**Figure 6:**
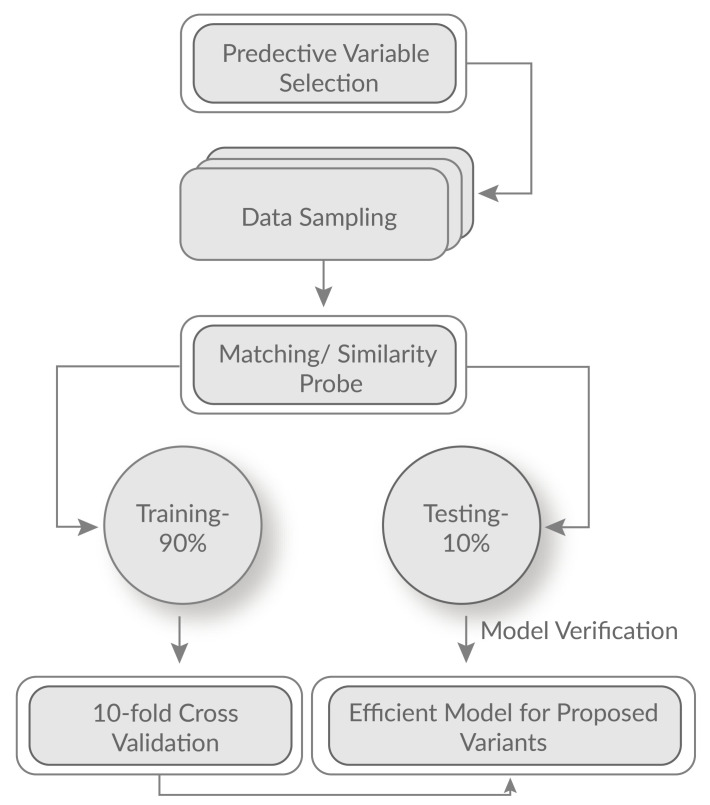
Optimal perdition model for conducting the experiment.

In addition, we also chose different training and testing options for study. Based on training and testing ratios of 80:20, the accuracy was observed 92.6% and 84.34%, for training and testing respectively. The 70:30 ratios produce the accuracy of 69.3% and 81.30% respectively. Therefore, it shows the possible overfitting. We employed 90:10 ratios that demonstrate 99.65% and 94.6% for training and testing respectively. Furthermore, 10-fold cross-validation is used because it helps to divide the samples into training data sets to train the model and use a test data set to verify it. Figure 6 shows the efficient perdition model for the support of proposed variants.

### 3.5. Separability

The spectral separability between selected ROI pairs is computed for a given input file [27]. The value of the separability between each pair is from 0 to 2, and values greater than 1.9 indicate good separability [29]. Good separability means a good ability for computers to perform machine learning, which usually leads to high accuracy.

The separability formula can be established in the form of $S{⨂}_{k}S$, where *S* is the finite separability for the ground field *k*. Let *p* be a primitive element for the *S* over *k*; thus, $p=p_{1},p_{2},...,p_{n}$ are the conjugate of the splitting field ω for the lowest polynomial of *p*. Let M(p) be the matrix, written as

(6) $$M\left(p\right)=\left(\begin{array}{c} 1p_{1}p_{1}^{2}p_{1}^{3}...p_{1}^{n-1}\\ (1p_{2}p_{2}^{2}p_{2}^{3}...p_{2}^{n-1}\\ {}***\\ 1p_{n}p_{n}^{2}p_{n}^{3}...p_{n}^{n-1} \end{array}\right)$$

This describes the linear operator in $\omega^{n}$, which is indexable by the presumed separability.

### 3.6. Supervised Calculation

After choosing ROIs and computing their separability, the image can be classified using supervised classification with the existing algorithms. The next section introduces the mathematical principles of those three classification algorithms.

## 4. Mathematical Modeling and Characterization of MDC and MLC

### 4.1. Minimum Distance Classification

**Figure 7:**
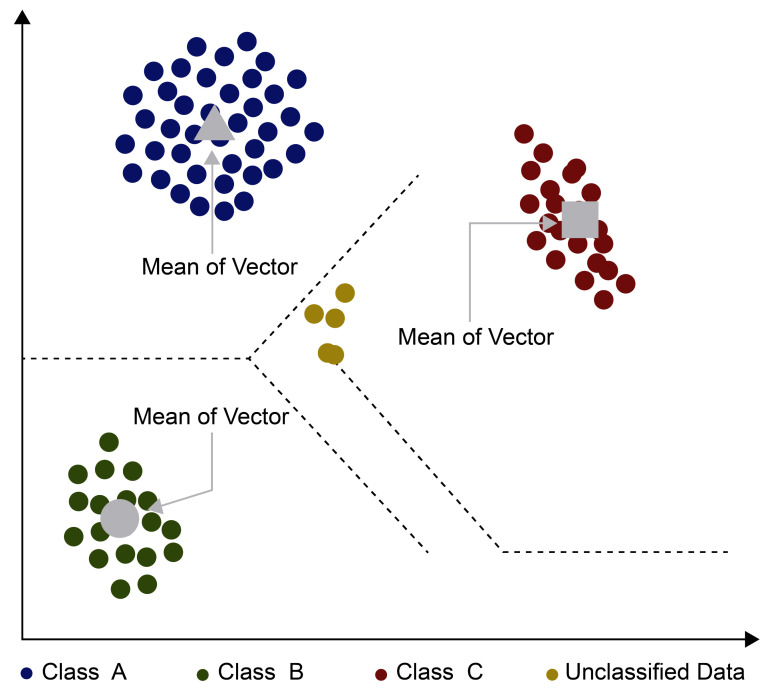
Principle of minimum distance classifier.

The minimum distance classification algorithm first calculates the mean vectors and draws the decision boundary for each class as shown in Figure 7. The pixels are then classified to the nearest class according to the decision boundary [30]. In the rectangular coordinate system, the coordinate of the mean vector of each class is calculated as the average of the coordinates of the entire pixel in that class:

(7) $$m_{i}=\frac{1}{N_{i}}\sum_{x\in c_{i}}x\;for\;i=1,2,...,M$$

where *M* is the number of classes and $N_{i}$ is the number of training data from the class $c_{i}$.

For any pixel *x* to be classified, the algorithm first calculates its Euclidean distance between every given classes, given by

(8) $$d_{i}\left(x\right)=||x-m_{i}||\;for\;i=1,2,...,M$$

If $x=\left(p_{1},q_{1}\right)\;m_{i}=\left(p_{2},q_{2}\right)$. It is then equal to

(9) $$D_{i}\left(x\right)=\sqrt{{\left(p_{1}-p_{2}\right)}^{2}+{\left(q_{1}-q_{2}\right)}^{2}}$$

However, for computers, it is usually convenient and efficient to perform matrix calculation. In that sense, for column vectors *x* and $m_{i}$, the distance between *x* and m(i) is

(10) $$D_{i}{\left(x\right)}^{2}={\left(x-m_{i}\right)}^{T}\left(x-m_{i}\right)=x^{T}x-x^{T}m_{i}-m_{i}^{T}x+m_{i}^{T}m_{i}$$

Because for a given pixel *x*, $x^{T}$ and *x* are the same as the distance to the mean vectors of every class; also $x^{T}$$m_{i}=m_{i}^{T}x$, which is equivalent to computing and defined by

(11) $$d_{i}{\left(x\right)}^{2}=x^{T}m_{i}-\frac{1}{2}m_{i}^{T}m_{i}\;for\;i=1,2,...,M$$

Finally, the decision boundary that separates class $c_{i}$ and $c_{j}$ is given by

(12) $$d_{i}{\left(x\right)}^{2}-d_{i}{\left(x\right)}^{2}=0\;for\;every\;i\neq j$$

The pixel *x* is classified as class $c_{i}$ if it falls into the area of the intersection of all the decision boundaries from that class.

### 4.2. Maximum Likelihood Classification

**Figure 8:**
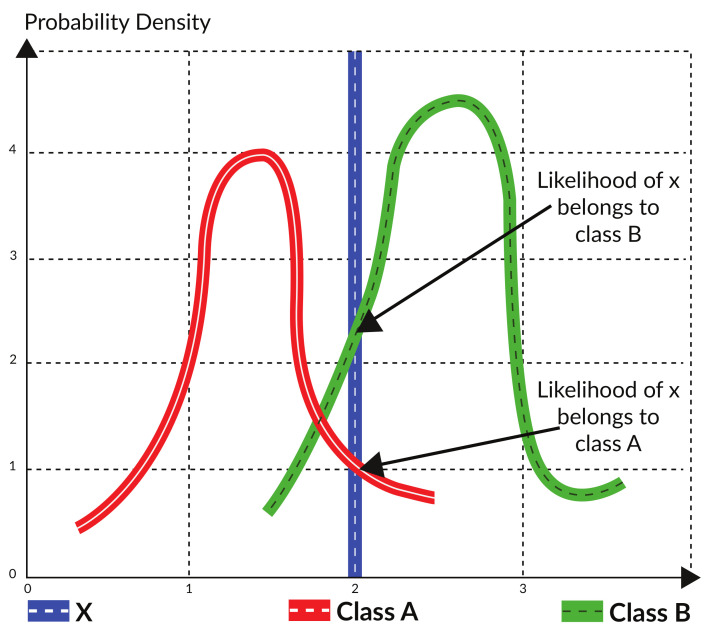
Principle of maximum likelihood classifier.

As depicted in Figure 8, the MLC calculates the probability that a given pixel belongs to a specific training class *n* based on the assumption that the statistics for each training class in each band are typically distributed. We then classify the pixel into the class with the maximum probability [30]. The accuracy of MLC and the contending method is shown in Table 2.

The basic principle of MLC is based upon the Bayes theorem, which states that an a posteriori distribution $P(c_{i}|x)$ can be calculated by the prior probability $P(x|c_{i})$.

(13) $$L\left(x\right)=P\left(c_{i}|x\right)=P\left(x|c_{i}\right)\times P\left(c_{i}\right)/P\left(x\right)$$

The pixel *x* will be classified into class $c_{i}$ if $P(c_{i}|x)$ is the largest among all the training classes:

(14) $$x\in c_{i}\;if\;P\left(c_{i}|x\right)>P\left(c_{i}|x\right)\;for\;all\;i\neq j$$

In the right hand of the function, $P(x|c_{i})$ shows, in a given training class $c_{i}$, the probability that a pixel appears in the position of *x*. $P(c_{i})$ is the probability that class $c_{i}$ occurs in the study area, which is a priori information, and P(x) is the probability that pixel *x* is observed, which can be written as

(15) $$P\left(x\right)=\sum_{i=1}^{M}P\left(x|c_{i}\right)\times P\left(c_{i}\right)$$

It is obvious that P(x) is constant within every training class, and that $P(c_{i})$ is a priori information that is usually not considered in the classification. This assumption, however, will have detrimental effect on the accuracy in some cases, as discussed later. Thus, the above rule is equivalent to

(16) $$x\in c_{i}\;if\;P\left(x|c_{i}\right)>P\left(x|c_{i}\right)\;for\;all\;i\neq j$$

As stated before, MLC assumes that the distribution of the data within a given class $c_{i}$ obeys a multivariate normal distribution [30]. The probability density function with a normal distribution in an n-dimensional space is given by

(17) $$f\left(x\right)={\sqrt{{\left(2\pi \right)}^{n}|S_{k}|}}^{-1}exp\left[-\frac{1}{2}{\left(x-\mu_{k}\right)}^{T}S_{k}^{-1}\left(x-\mu_{k}\right)\right]$$

where $S_{k}$ is the covariance matrix of the M bands in the *k*th class. However, for computers, exponential computation always has a large time complexity, which may take a large amount of time. So, some simplification is needed for future computation. First, the exponent can be removed by a logarithm, since the log is monotonically increasing in its domain.

(18) $$G_{i}\left(x\right)=Ln\;P\left(x|c_{i}\right)$$

(19) $$=Ln{\sqrt{{\left(2\pi \right)}^{n}|S_{c_{i}}|}}^{-1}-\frac{1}{2}{\left(x-\mu_{c_{i}}\right)}^{T}S_{c_{i}}^{-1}\left(x-\mu_{c_{i}}\right)$$

(20) $$=Ln{\sqrt{{\left(2\pi \right)}^{n}|S_{c_{i}}|}}^{-1}-\frac{1}{2}{\left(x-\mu_{c_{i}}\right)}^{T}S_{c_{i}}^{-1}\left(x-\mu_{c_{i}}\right)$$

Then, if the features we choose are identical, and the features in each dimension are mutually independent, the covariance matrix of the M bands in every class will be the same, as given by

(21) $$G_{i}\left(x\right)=-\frac{n}{2}Ln\left(2\pi \right)-\frac{1}{2}Ln\left(|S|\right)-\frac{1}{2}x^{T}S^{-1}x+x^{T}S^{-}1\mu_{c_{i}}-\frac{1}{2}\mu_{c_{i}}^{T}S^{-1}\mu_{c_{i}}$$

Finally, since the first three terms—$\frac{n}{2}Ln\left(2\pi \right)-\frac{1}{2}Ln\left(|S|\right)-\frac{1}{2}x^{T}S^{-1}x$—are the same in each class, the function can be simplified as follows:

(22) $$g_{i}\left(x\right)=x^{T}S^{-}1\mu_{c_{i}}-\frac{1}{2}\mu_{c_{i}}^{T}S^{-1}\mu_{c_{i}}$$

$x\in c_{i}$ if $g_{i}\left(x\right)>g_{j}\left(x\right)$ for all i≠j.

### 4.3. Novel Working Principles of SVM-RBF and SVM-Linear

**Figure 9:**
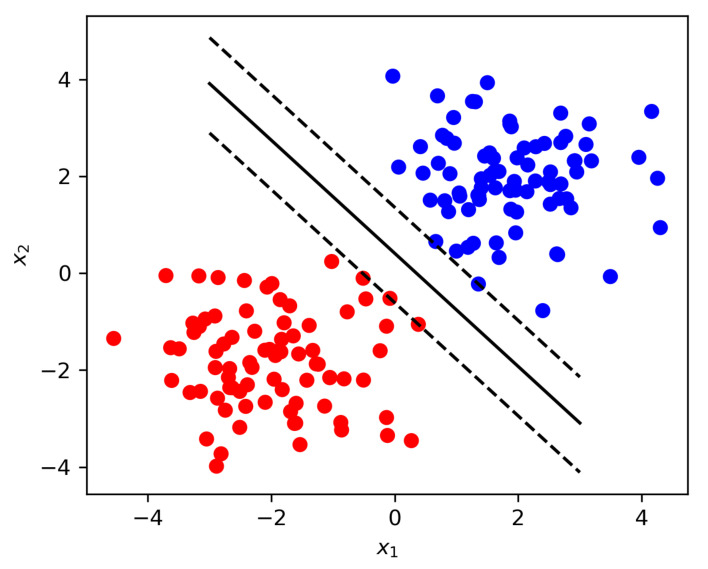
Hyperplane formation for the novel SVM technique.

We define the new SVM-RBF and SVM-Linear as the aggregation variants that can be used for both classification and regression. Unlike traditional statistic-based parametric classification algorithms, the SVM-RBF and SVM-Linear variants are non-parametric, and since the SVM is one of the most popular non-parametric machine learning algorithms, but it degrades the performance with many samples, Thus, novel SVM-RBF and SVM-Linear variants improve the change detection accuracy and efficiency. Also, they do not require to make any assumptions regarding the distribution of data. When the data cannot be separable as SVM-Linear, then a nonlinear improved SVM-RBF uses functions to minimize the computational load. This process is known as the kernel-trick. The polynomial kernel and Gaussian kernel approaches are popular. Let us assume that n-dimensional data points $a_{i}\in Z^{m}$(i=1,...,M) correspond to either class 1 or class 2; then, the associated class labels take $x_{i}=1$ and $x_{i}=-1$ for classes 1 and 2, respectively. If data are linearly separable, then the SVM-Linear is identified as the problem for finding the discriminant function.

(23) $$D\left(f\right)=\Upsilon^{T}+bi$$

where Υ is the normal vector for hyperplane separation; bi is the bias. The SVM-Linear reduces the distance to the adjacent data point depicted in Figure 9. The margin distance, “*r*”, is given by

(24) $$r=\frac{1}{\left\|\;r\;\right\|}$$

The hyperplane has the nearest data points, which are called support vectors, Thus, the support vectors generate the discriminant function. The reduction problem of the distance margin can be formulated as the reduction problem of ${\left\|\;\Upsilon \;\right\|}^{2}$, which is calculated as

(25) $$R\left(Q(\Upsilon ,bi)\right)=\frac{1}{2}{\left\|\;\Upsilon \;\right\|}^{2}$$

Subject to $x_{i}\left(\Upsilon^{T}a_{i}+bi\right)\geq 1\left(i=1,...,M\right)$.

The Lagrange function ′L′ is used to obtain the dual problem

(26) $$L\left(\Upsilon ,bi,p\right)=\frac{1}{2}{\left\|\;\Upsilon \;\right\|}^{2}-\sum_{i=1}^{M}p_{i}\left\{x_{i}\left(\Upsilon^{T}a_{i}+bi\right)-1\right\}$$

where $p=(p_{1},...,p_{M})$ is the Lagrange multiplier. Therefore, the dual problem *d* can be calculated as

(27) $$d\left(Q\left(p\right)\right)=\sum_{i=1}^{M}p_{i}-\frac{1}{2}\sum_{i,j=1}^{M}p_{i}p_{j}x_{ji}x_{j}y_{i}^{T}y_{j}$$

The above equation is a hard-margin SVM subject to

(28) $$\sum_{i,j=1}^{M}p_{i}x_{i}=0,p_{i}\geq 0\left(i=1,...,M\right)$$

**Figure 10:**
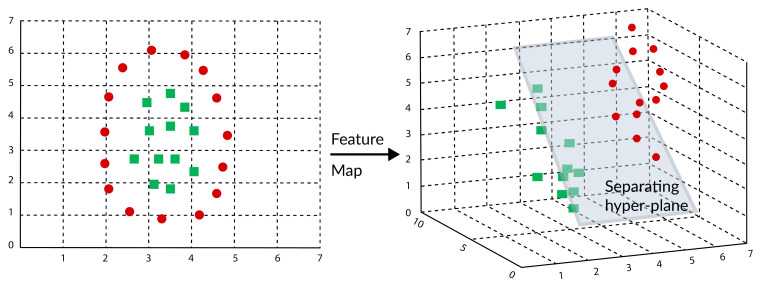
Feature mapping and hyperplane.

In a linear separable case, the goal of learning in the SVM-Linear is to find a linear hyperplane, as depicted in Figure 9, that not only separates samples from different classes but also has the maximum margin. The margin indicates the distance between the hyperplane and the nearest training samples (support vectors). For binary classification, if the training data with m number of samples are represented as $\left\{X_{i},y_{i}\right\}$, where $X\in R^{n},i=1,2,...\;k,$ and y∈0,1, while y=0 indicates class 1 and y=1 indicates class 2, the hyperplane is formed to find the optimal θ depicted in Figure 9 that minimizes the cost function. Additionally, feature mapping and the hyperplane are further depicted in Figure 10.

(29) $$J\left(\theta \right)=C\sum_{i=1}^{m}\left[y^{\left(i\right)}cost_{1}(\theta^{T}X^{\left(i\right)})+(1-y^{\left(i\right)})cost_{2}(\theta^{T}X^{\left(i\right)})+\frac{1}{2}\right]\sum_{i=0}^{n}\theta_{j}^{2}$$

where

$cost_{1}=logh_{\theta }\left(x\right)=log\frac{1}{1+e^{-\theta^{T}x}}$

$cost_{1}=log\left(1-h_{\theta }\left(x\right)\right)=log1-\frac{1}{1+e^{-\theta^{T}x}}$

So, θ is calculated according to the following rule.

$\theta^{T}X^{\left(i\right)}\leq -1$ to minimize J(θ) when y=0

$\theta^{T}X^{\left(i\right)}\geq 1$ to minimize J(θ) when y=1

The new data *x* are classified into either class y=0 or y=1 according to the hyperplane. In the non-linear separable case, the SVM-RBF uses a pre-selected nonlinear mapping function to map input variables to a high-dimensional feature space, constructing the optimal classification hyperplane in the space. The SVM-RBF and SVM-Linear Variants will then find the hyperplane that has the same features as the straight line in the previous case. There are four kernel functions that are commonly used, but we improved and used the two: the linear and radial basis function variants, also known as the Gaussian variants in our research.

Let Ψ and ǵ be the both parameters for the improved SVM-Linear $SVM_{l}$. Thus, the improved SVM-Linear variant can be stated as:

(30) $$SVM_{l}=f\left(x\right)=\sum_{i=1}^{N}\Psi \cdot \forall \omega \left(a,a_{i}\right)+ǵ$$

Therefore, the improved SVM-RBF $SVM_{RBF}$ consists of σ and λ parameters. As parameter σ is applied for the function-execution. Whereas, the parameter λ is a highly important that creates the trade-off between estimated function and the minimum fitting error. Thus, the improved SVM-RBF can be determined as:

(31) $$SVM_{RBF}=\forall \omega \left(a,a_{i}\right)=exp\left(-\frac{1}{\sigma^{2}}||a,a_{i}|{|}^{2}\right)\times \lambda$$

where $\forall \omega (a,a_{i}$: attributes of improved SVM-Linear; and ∀ω: variant function.

SVM-RBF possesses the Non-linearity features that achieve two conditions. First, the SVM-RBF should be symmetric, and the second must have a capability to guarantee the space determination with the real-world problem (the pairwise integration capability). Thus, the first and second conditions are given in Equations (32) and (33).

(32) ∀ω((a,p)=(φ(a)·φ(p)))

(33) $$\forall \omega \left(a,p\right)-\{\varphi (a)\cdot \varphi (p)\}=\{(a)\cdot \varphi (p)-\forall \omega (a,p)\}$$

The SVM-RBF has a capability by combining binary classifiers using the technique of one versus all (OVA). In a *k* classification problem, the one versus all method produces one binary classifier for every class, with the samples of that class being *y* = 1 and all samples of the other (k−1) classes being y=0. Thus, taken together, there will be *k* binary classifiers. To classify new data *x*, all the *k* binary classifiers will run and *x* will be classified into class *i* that returns the highest confidence and maximum classification value [31].

### 4.4. Fault-Tolerance Process of SVM-RBF and SVM-Linear

Let us consider the probability ‘Pr’ that is estimated when a sensor/actor are not faulty, as given by

$$P_{r}(B_{i}=0||Sre=0,T_{g}=0)$$

where $B_{i}$ is a binary variable with decoder value, $S_{re}$ is a sensor-reading and $T_{g}$ is the ground truth.

(34) $$P_{r}(B_{i}=0||Sre=0,T_{g}=0)=\sum_{k=0}^{N}P_{r}(B_{i}=0||Sre=0,T_{g}=0),\gamma \left(0,k\right)=\sum_{k=0}^{N}P_{c}\sigma$$

where γ(0,k) is the *K* value of the sensor/actor nodes that have same reading, $P_{c}$ is the conditional probability and σ represents not faulty neighbors.

Similarly, we can determine the expressions for conditional probabilities.

(35) $$P_{r}(B_{i}=\beta ||Sre=\beta ,T_{g}=\beta )=1-P_{r}(B_{i}=-\beta ||Sre=\beta ,T_{g}=\beta )=\sum_{k=0}^{N}P_{c}\sigma$$

(36) $$P_{r}(B_{i}=-\beta ||Sre=-\beta ,T_{g}=\beta )=1-P_{r}(B_{i}=\beta ||Sre=-\beta ,T_{g}=\beta )=\sum_{k=0}^{N}P_{c}\sigma -k$$

Thus, the expected number of decoded errors β can be obtained by disregarding values for $S_{re}$:

(37) $$\beta =P_{r}{\left(B_{i}=1||T_{g}=0\right)}_{\delta }+P_{r}{\left(B_{i}=0||T_{g}=0\right)}_{\psi }=\left(1-\sum_{k=0}^{N}P_{c}\left(\sigma -\tau -k\right)\right)tk$$

where β is the average number of errors after decoding, δ is the number of other nodes, ψ is the nodes in the affected region, τ is the expected faulty nodes and tk is the total deployed nodes in the network.

Therefore, the reduced errors can be obtained as

$$\frac{\left(tk\cdot P_{r}-\beta \right)}{tk\cdot P_{r}}$$

Thus, we can show explicitly β that the average number of corrected faults μ in sensor/actors can be obtained by combining the conditional probabilities of Equations (38) and (39):

(38) $$\mu =\left(1-\sum_{k=0}^{N}P_{c}\left(\sigma -k\right)\right)tk\cdot P_{r}$$

The number of uncorrected faults can be given by

(39) $$\mu^{-}=\left(1-\sum_{k=0}^{N}P_{c}\left(\sigma -k\right)\right)tk\cdot P_{r}$$

where $\mu^{-}$: uncorrected faults

**Figure 11:**
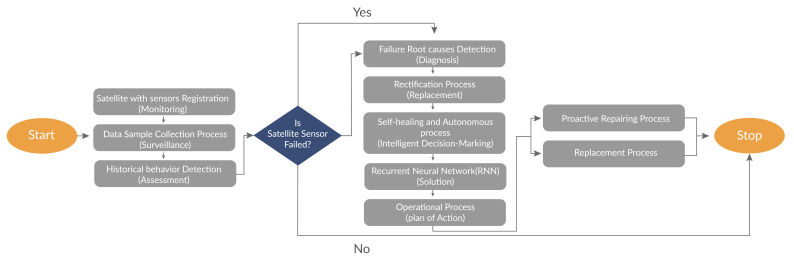
Fault Diagnosis/Tolerance process for Satellite Sensor for the RS.

The fault diagnosis/tolerance is depicted in Figure 11. If the satellite sensor fails to function, then there is a need to diagnose immediately. Thus, the framework used in [32] has been used to support the proposed variants to avoid the possible delay due to the failure of the sensor.

## 5. Experimental Results

This section presents the experimental results in terms of the experimental setup and performance results for the SVM-RBF and SVM-Linear, NDCI [8], HSRS [19], SCMask R-CNN [17], CIAs [18], KCA [21], AOPC [22], MLC [33] and MDC [34].

### 5.1. Experimental Setup

Most of the experiments were implemented on the system with Quad-Core Processor 3.3 GHz 4 Core, and 24-GB memory using ENVI5.3. We also made some minor redevelopments using IDL programming in order to separate the training and test set. We also implemented the SVM-RBF and SVM-Linear on MATLAB with the help of libsvm in order to do cross-validation to optimize the parameters. The remaining parameters are given in Table 3. Optical remote sensing and thermal remote sensing have been applied to obtain the result. The former type is used for obtaining the reliability, time complexity, accuracy, and fault-tolerance, but later is used only with fault-tolerance.

### 5.2. Performance Metrics

Performance metrics are delineated as figures and data representative of the capabilities of parametric and non-parametric algorithms and their overall performance. Based on the obtained data, the results are revealed in the form of graphs to view the measurements of the following metrics as a source of comparison:

- Accuracy;
- Time complexity;
- Fault tolerance;
- Reliability.

#### 5.2.1. Accuracy

In our assessment, we use two of the most common criteria used for accuracy estimation. The simplest criteria for the accuracy of classification result is the overall accuracy ‘$A_{O}$’, which represents how well the image area is correctly classified, given by

(40) $$A_{O}=\sum \frac{X_{ii}}{T}\;for\;i=0,1,...,N$$

However, the overall accuracy cannot provide specific information about the accuracy of each individual class and neglects severe partial errors [35]. In order to take the accuracy of each individual class into consideration, the Kappa coefficient which takes both the overall $A_{O}$ and partial accuracy $P_{0}$ into consideration is introduced to evaluate the accuracy of the classification result of the two algorithms.

(41) $$K=\frac{\left(P_{O}-P_{e}\right)}{1-P_{e}}$$

(42) $$P_{O}=\sum \frac{x_{i+}x_{+i}}{T^{2}}\;for\;i=0,1,...,N$$

Kappa coefficients are used to test the consistency of ground data and classified data, where K=1 means that all pixels are correctly identified [36].

**Figure 12:**
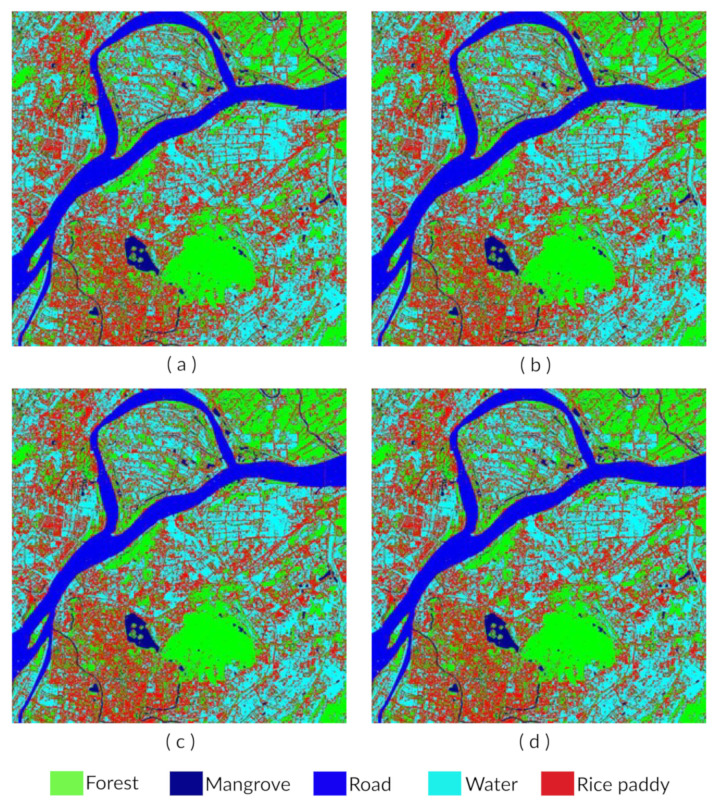
Four images representing the classification results with four classifiers. (**a**) Classification result with the MDC; (**b**) Classification result with the MLC; (**c**) Classification result with the SVM using a linear kernel; (**d**) Classification result with the SVM using a default RBF.

In the training set, we can see that, for parametric classifiers, the MLC, depicted in Figure 12b, performs much better than the MDC, depicted in Figure 12a, having a significantly higher overall accuracy and Kappa coefficient of 94.00% and 0.92 than the values of 80.58% and 0.75 for the MDC, respectively. The MDC is severely affected by the classes with similar spectral behaviors due to its simple mathematical principle. The maximum likelihood classification algorithm, however, is relatively less affected due to its complex mathematical principle of taking both mean vectors and covariance into consideration.

The introduced non-parametric SVM, however, does neither increases the overall accuracy nor the Kappa coefficient considerably compared with the MLC in the training set. The different kernels and parameters in the SVM do not seem to change the performance either. The overall accuracy and Kappa coefficient all remain around 94.5% and 0.93, respectively. Thus, some researchers stop here, reaching the conclusion that the MLC is suitable for RS classification.

However, in the test set, the accuracy and Kappa coefficient of ML declines dramatically to 80% and 0.74, respectively. The accuracy is observed to steadlily increase when we introduce the SVM linear variant to 81.33%, as depicted in Figure 12c. Since the data are more likely to be non-linear separable, the accuracy and Kappa coefficient keep increasing considerably to 85.40% and 0.80 when we implement the SVM-RBF using the default parameter (C = 100, r = 0.33), as depicted in Figure 12d. Finally, the accuracy and Kappa coefficient reach 89.32% and 0.84, respectively, when we use the optimal parameter found in cross validation.

**Figure 13:**
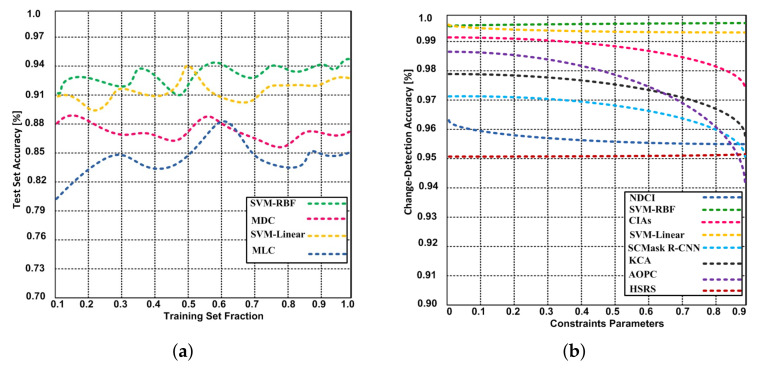
Accuracy. (**a**) Accuracy of the SVM-RBF, SVM-Linear, MLC and MDC approaches with different training set fractions. (**b**) The relationship between constraints parameters and change-detection accuracy of proposed SVM-RBF, SVM-Linear and competing algorithms.

From the accuracy estimation, it is clear that the MLC is much more accurate than the MDC both for known and unknown data. The introduction of the SVM-RBF and SVM-Linear and parameter optimization does improve the accuracy but not considerably compared with the MLC on known data. However, the increase is remarkable with unknown data. Moreover, from the classification result image, when can see that the distribution of data with both parametric classifiers—especially the MDC—tend to be fragmented, which may result in “pepper and salt” noise [37]. The distribution in the result of the SVM is quite compact, which corresponds well to the real case [12]. It is validated that the SVM-RBF and SVM-Linear obtains better generalization performance on unknown data while the traditional parametric classifier is severely affected by the problem of overfitting due to disturbing information. However, the difference in accuracy is within 1% for the training set. However, our results for the test set indicate that SVM with the RBF kernel produces a higher level of accuracy compared to SVM with a linear kernel, the MLC and the MDC, as depicted in Figure 13. We can conclude that the SVM-RBF and SVM-Linear obtains good generalization performance on anonymous data and is more suitable for practical use than traditional parametric classifiers. However, there is a need for further research with respect to the implementation of the SVM-RBF and SVM-Linear on RS image classification [7,18]. Firstly, the probabilities of SVM-RBF and SVM-Linear are much higher than MLC and MDC, especially in the cross validation for the parameter optimization of Υ and C. As a future study, the parameter optimization may be accomplished in other more efficient ways. However, during the inference phase, all of the three classifiers do not take large amounts of time. Furthermore, the training of the classifier is only performed once. Secondly, though the accuracy of SVM on unknown data was significantly improved compared to traditional parametric classifiers, it was still less than 90%. Random sampling was adopted to accomplish the accuracy analysis objectives, consisting of computing the uncertainty estimates and unbiased accuracy. Thus, the sample size $S_{s}$ can be calculated as

(43) $$S_{s}=\frac{\mu A_{o}\left(1-A_{o}\right)}{C_{i}}$$

where μ is a standard normal distribution percentile; $A_{o}$ is the overall accuracy; and $C_{i}$ is the margin of error. The variance accuracy V(A) depends on the map proportion $M_{P}$ of each class that requires an individual cell probability $I_{p}$, which depends on the portion of the entire classified map.

(44) $$V\left(A\right)=\sum_{i=1}^{q}=I_{p}\frac{M_{P}-I_{p}}{M_{P}\times S_{C}}$$

where $S_{C}$ is the sample counts.

Furthermore, the change-detection accuracy of proposed SVM-RBF and SVM-Linear is identified and also compared with the state-of-the-art algorithms: NDCI, SCMask R-CNN, CIAs, KCA HSRS, and AOPC. The results confirm that 99.65% and 99.43% change-detection accuracy has been obtained with SVM-RBF and SVM-Linear respectively; whereas the NDCI, CIAs, SCMask R-CNN, KCA, HSRS and AOPC have obtained the change-detection accuracy 95.6%, 97.4%, 95.0%, 95.8%, 95.2% and 94.2% respectively. The AOPC and CIAs produced the lower accuracy due to higher sensitivity of the algorithms and extreme learning machine to noise. Deep learning methods such as R-CNN require large amount of data. CNN model has many parameters which need to be optimized during the training process. The data of remote sensing image is relatively small. Thus, deep learning methods face difficulty in learning very from such data.

#### 5.2.2. Time Complexity

**Figure 14:**
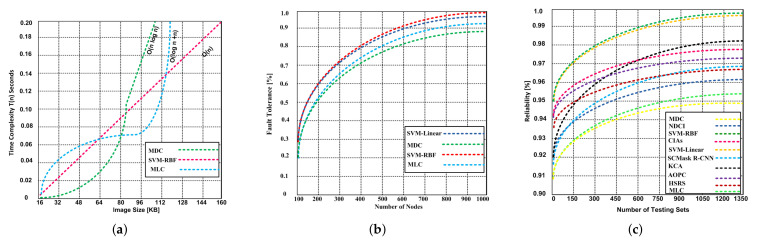
Time complexity, fault tolerance and reliability. (**a**) Time complexity of SVM-RBF, MDC and MLC; (**b**) Fault tolerance of SVM-RBF, MDC and MLC; (**c**) Reliability of SVM-RBF, SVM-Linear, NDCI, SCMask R-CNN, CIAs, KCA, HSRS, and AOPC and MDC and MLC classifiers.

The performance of an algorithm depends on the time complexity. The time complexity refers to the amount of time required to run as a task, signifying the input. In addition, the time complexity is measured by calculating the number of basic operations accomplished by the algorithm, and a basic operation takes a constant amount of time to execute. In Figure 14, we show the trend of the time complexity for parametric and non-parametric classification algorithms: the results show that the non-parametric classification algorithm SVM-RBF exhibits O(n) time complexity, whereas the MLC and MDC exhibit complexities of (log n+n) and O(n log n), respectively.

SVM-RBF shows the lowest time complexity compared to other parametric algorithms (MLC and MDC). The reason for the low latency is the use of new machine learning methods. On the other hand, parametric classifiers are based on traditional statistics. The time complexity of the three algorithms is measured using the recursive approach obtained by using Equation (45).

(45) $$T\left(N\right)=\left\{\begin{array}{cc} O(1) & \mathrm{if}\;n=1\\ at\left(\frac{n}{b}\right)+0\left(n\right) & \mathrm{if}\;n\;>\;1 \end{array}\right.$$

Table A1 (Appendix A) shows the time complexity of competing algorithms.

#### 5.2.3. Fault Tolerance

A system must have the ability to continue functioning without disruption when its components fail. Some of the algorithms implemented on those systems will experience a problem due to the fault tolerance process. As RS requires robust algorithms to cope with such a critical situation, the algorithms used in RS are of paramount importance, since the reliability of the result from RS depends heavily on the classification accuracy. Parametric classifiers based on traditional statistics have successfully been used in RS classification, but the accuracy is greatly impacted and rather constrained by the statistical distribution of the sensing data.

We determine the fault tolerance capability of three classifiers: the MDC, SVM-RBF, SVM-Linear and MLC. Thus, an experiment has been conducted that involved 1000 nodes deployed in the area of 400 × 400 square meters. Based on the testing results, it is observed that the SVM-RBF and SVM-Linear have a better fault tolerance capability compared to MDC and MLC, as depicted in Figure 14c. Two different types of experiments have been conducted. In First experiment, the result is obtained based on optical remote sensing. The result demonstrate that the SVM-RBF has 99.99% fault tolerance capability with 1000 nodes, whereas the MDC and MLC have 88.12% and 92.32%, respectively.

**Figure 15:**
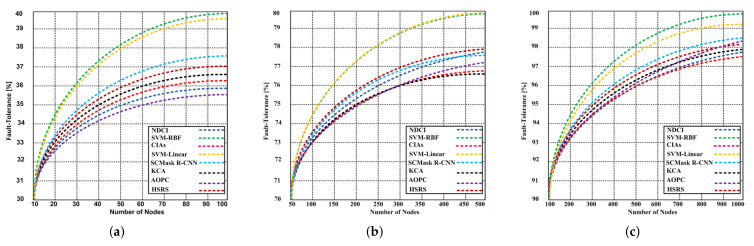
(**a**) Fault-tolerance of the proposed SVM-RBF and SVM-Linear variants and contending algorithm (NDCI, SCMask R-CNN, CIAs, KCA, HSRS, and AOPC) with maximum 100 nodes using thermal remote image sensing; (**b**) Fault-tolerance of the proposed SVM-RBF and SVM-Linear variants and contending algorithm (NDCI, SCMask R-CNN, CIAs, KCA, HSRS, and AOPC) with maximum 500 nodes using thermal remote image sensing; (**c**) Fault-tolerance of the proposed SVM-RBF and SVM-Linear variants and contending algorithm (NDCI, SCMask R-CNN, CIAs, KCA, HSRS, and AOPC) with maximum 1000 nodes using thermal remote image sensing.

In the second experiment, the result is shown by using thermal sensing. In this experiment, three different scenarios are generated and different numbers of the nodes are randomly distributed. If fewer nodes are deployed in the allocated region, then the fault-tolerance capacity is reduced. The proposed SVM-RBF and SVM-Linear are compared with SVM, NDCI, SCMask R-CNN, CIAs, KCA, HSRS, AOPC, MDC, and MLC classifiers. Additionally, the new public datasets have been used. When the maximum 100 nodes are deployed as depicted in Figure 15a, then the SVM-RBF and SVM-Linear produce better fault-tolerance capacity that is 39.92% and 39.73% respectively; whereas the contending algorithms produce reduced fault-tolerance capacity that is 35.53–37.78%. When the maximum 500 nodes are deployed as shown in Figure 15b, then SVM-RBF and SVM-Linear yield the same fault-tolerance capacity that is 79.88%; whereas the contending algorithms give 76.71–78.94% fault-tolerance. Finally, we increased the number of nodes up to 1000 to cover the entire region shown in Figure 15c. The results confirm that our proposed SVM-RBF, and SVM-Linear 99.92% and 99.29% respectively. On the other hand, the contending algorithms showed 97.48–98.51% fault tolerance. In these scenarios, one thing that is interesting to note that the proposed variants yield approximately 2% higher fault tolerance as compared to the contending algorithms.

#### 5.2.4. Reliability

If the classifiers work efficiently and all of the components of the devices operate and support properly, then the reliability $R_{al}$ is obtained as

(46) $$R_{al}=P_{r}\left\{\varepsilon (k(\tau )=1)\right\}\prod_{k=0}^{n}P_{r}\left\{k(\varepsilon )=1\right\}=\prod_{k=0}^{n}R_{k}\times \omega \left(t\right)$$

where k(ε) is the functioning probability of either actor/sensor node and $R_{k}\times \omega \left(t\right)$ is the reliability of the total components used in the network.

The reliability of algorithms is highly important for RS. In this experiment, the reliability of SVM-RBF, SVM-Linear, NDCI, SCMask R-CNN, CIAs, KCA, HSRS, AOPC, MDC, and MLC classifiers has been examined. Again, based on the results of testing sets, the SVM-RBF and SVM-Linear outperform the other contending algorithms. The result depicted in Figure 14c shows that the SVM-RBF and SVM-Linear show 99.92% reliability, while the contending algorithms exhibit 93.8–98.2% reliability. The MDC and MLC produce the lower 93.8% and 95.4% reliability respectively.

## 6. Conclusions and Future Analysis

This section reiterates the objectives and summarizes the key findings for the reader. Additionally, it provides the directions for the future analysis.

### 6.1. Conclusions

With the wide use of RS technologies, the accuracy of classifiers in RS classification has become increasingly critical. In this paper, the improved SVM-RBF and SVM-Linear variants have been introduced to improve the test set accuracy, detection accuracy, reliability, time complexity, and fault-tolerance. Land-use classification is created for sensing images taken by Landsat-8 satellite for the area of Nanjing, China, and the Tanintharyi region located in southern Myanmar. Two types of images were used for obtaining the results: Optical and thermal remote images for different types of data sets. The analysis demonstrates that the proposed SVM-RBF and SVM-Linear variants are useful for RS. The paper involves the following conclusion.

- The proposed variants are capable to address mask generation, cross-validation, ranking. change classification/No-change classification, underfitting, and overfitting.
- The SVM-RBF and SVM-Linear are compared with the state-of-the-art algorithms (NDCI, SCMask R-CNN, CIAs, KCA, HSRS, and AOPC from the change detection accuracy, and reliability standpoint. The proposed SVM-RBF and SVM-Linear have obtained 99.65% and 99.43% change-detection accuracy respectively; whereas the NDCI, CIAs, SCMask R-CNN, KCA, HSRS, and AOPC have obtained the change-detection accuracy 95.6%, 97.4%, 95.0%, 95.8%, 95.2%, and 94.2% respectively.
- The SVM-RBF and SVM-Linear variants performed well on the training data with an overall accuracy of around 94% and Kappa coefficient around 0.92 which is much higher than the MDC and MLC algorithms.
- The SVM-RBF has 99.99% fault tolerance capability with 1000 nodes, whereas the MDC and MLC have 88.12% and 92.32% respectively.
- The SVM-RBF and SVM-Linear show 99.92% reliability, while the contending algorithms exhibit 93.8–98.2% reliability. The MDC and MLC produce the lower 93.8% and 95.4% reliability respectively.
- The SVM-RBF produces O(n) time complexity that is reasonable with remote image sensing.
- SVM-RBF obtains good generalization performance on unknown data and is more suitable for practical use than traditional parametric classifiers.
- The time spent by the SVM-RBF is much lower than the MLC and MDC, especially in the cross-validation for parameter optimization.

### 6.2. Future Work

As a future study, parameter optimization may be accomplished in other, more efficient ways. Secondly, though the accuracy of the SVM-RBF on anonymous data significantly improved. However, improved SVM-RBF and SVM-Linear variants should integrate and leverage the features from the Recurrent Convolutional Neural Network to further increase. Furthermore, we will try to focus on the security of the proposed variants (SVM-RBF, SVM-Linear). The adversarial examples add little noise to the original image that leads to misclassification. We will try to design a restricted adversarial example solution based on RNN. The localization vulnerability is another big issue for the RS that leads to possible attacks. As a result, these localization problems cause service interruption. We will try to formulate the localization vulnerability detection and prevention processes.

## Figures and Tables

**Table 1:** Showing characteristics/Features of contending algorithms for the land cover and land use.

Characteristics/Features	NDCI [8]	R-CNN [17]	CIAs [18]	HSRS [19]	KCA [21]	AOPC [22]	Proposed Method
Segmentation/Preprocessing	No	Yes	Yes	No	Yes	No	Yes
Separability	No	No	Yes	No	No	Yes	Yes
Ranking Classification	Yes	Yes	No	Yes	No	No	Yes
Change Classification	Yes	No	Yes	Yes	Yes	Yes	Yes
No-Change Classification	No	No	No	Yes	No	No	Yes
Image Classification	Yes	No	Yes	Yes	Yes	Yes	Yes
Visual interpretation and Field Verification	No	Yes	No	Yes	No	No	Yes
Feature Mapping	No	Yes	No	Yes	Yes	No	Yes
Dealing with unlabeled samples	No	No	No	Yes	No	Yes	No
Deal with underfitting and Overfitting	No	No	No	No	Yes	Yes	Yes
Addressing Generalization Problem	No	No	Yes	No	No	No	Yes
Forest Detection	No	No	Yes	Yes	Yes	Yes	Yes
Bare Soil Ground Detection	Yes	No	No	Yes	Yes	No	Yes
Water Detection	No	No	Yes	No	Yes	No	Yes
Urbanization Region Detection	Yes	Yes	No	Yes	Yes	Yes	Yes
Cross validation Process	No	Yes	No	No	No	Yes	Yes
Mask Generation Process	No	Yes	No	Yes	No	-	Yes
Change Detection Accuracy	95.6%	95.1%	97.4%	95.2%	95.8%	94.2%	SVM-RBF = 99.65%,
							SVM-Linear = 99.43%

**Table 2:** Accuracy assessment of the classifiers on the test set.

Classifier/Criteria	Test Accuracy	Test Kappa Coefficient
MDC	72.82	0.64
MLC	80.03	0.74
SVM-Linear	81.33	0.75
Default SVM-RBF (C = 100, r = 0.33)	85.40	0.80
Improved SVM-RBF	89.32	0.84

**Table 3:** Parameters used to conduct the experiments.

Parameters	Values
MDC	72.82
Sensing time	2.5 milliseconds
Sensing samples	250
Kernel functions	Linear and RBF kernels
Training observation	1000
Testing observation	6000
Source of sensing Images	Landsat 8
Cross-validation	5

## Data Availability

The data that supports the findings of this research is publicly available as indicated in the references.

## Abbreviations

The following abbreviations and nomenclatures are used in this manuscript:

RS	Remote sensing
SVM	Support vector machine
MCD	Minimum distance classification
MLC	Maximum likelihood classification
ASTER	Advanced spaceborne thermal emission and reflection
TM	Thematic mapper
DT	Decision tree
RBF	Radial basis function
ROI	Region of interest
IDL	Interactive data language
ENVI	Environment for visualizing images
OVA	One versus all
CNN	Convolutional neural network
NA	Not applicable
*M*	Number of classes
γ	Kernel parameter to determine how many nearby samples the support vector will
	consider
*C*	Penalty factor used for regularization
$N_{i}$	Number of training data
$c_{i}$	Class
*x*	Pixel
$D_{i}\left(x\right)$	Euclidean distance
$P(c_{i}|x)$	A posteriori distribution
$P(x|c_{i})$	Prior probability
$P(c_{i})$	The probability that class $c_{i}$ occurs in the study area
P(x)	The probability that pixel *x* is observed
f(x)	The probability density function of normal distribution in an n-dimensional space
$S_{k}$	The covariance matrix of the *M* bands in *k*th class
$A_{O}$	Overall accuracy
$P_{0}$	Partial accuracy
Pr	Probability estimated when sensor/actor are not faulty
$B_{i}$	Binary variable with decoder value
$S_{re}$	Sensor-reading
$T_{g}$	Ground truth
γ(0,k)	*K* of the sensor/actor nodes that have same reading
$P_{c}$	Conditional probability
σ	Not faulty neighbors
β	Average number of errors after decoding
δ	Number of other nodes
ψ	Nodes in the affected region
τ	Expected faulty nodes
tk	Total deployed nodes in the network
μ	Average number of corrected faults
$\mu^{-}$	Number of uncorrected faults
$R_{al}$	Reliability
k(ε)	Functioning probability of either actor/sensor node
$R_{k}\times \omega \left(t\right)$	Reliability of total components used in the network

## Appendix A

**Table A1:** Time complexity for contending algorithms.

Methods	Time Complexity
SVM-RBF	$T\left(n\right)=at\left(\frac{n}{b}\right)+O\left(n\right)$
	Problem consists of finite set of inputs, but its computation time linearly increases. Thus,
	$T\left(n\right)=t\left(\frac{n}{2}\right)+O\left(n\right)$
	$T\left(n\right)=t\left(\frac{n}{n}\right)+O\left(n\right)$
	T(n)=t+O(n)
	Where *t* is ignored; therefore
	T(n)=O(n)
MDC	$T\left(n\right)=at\left(\frac{n}{b}\right)+O\left(n\right)$
	Where problem is divided into two parts with same size. However, the algorithm is infinite. Thus.
	$T\left(n\right)=2t\left(\frac{n}{2}\right)+O\left(n\right)$
	$T\left(n\right)=2t\left(\frac{n}{2}\right)+O\left(n\right)$
	$\left(n\right)=4t\left(\frac{n}{4}\right)+n+n$
	$T\left(n\right)=4t\left(\frac{n}{n}\right)+2n$
	T(n)=4t+2n
	T(n)=O(kn)
	$T\left(n\right)=O\left(\log_{nn}\right)$
	Where $k=\log_{n}$
	$T\left(n\right)=O\left(n\log_{n}+n\right)$
MLC	$T\left(n\right)=at\left(\frac{n}{b}\right)+O\left(n\right)$
	Problem consists of finite set of inputs, but computation complexity remains constant *n*
	$T\left(n\right)=t\left(\frac{n}{2}\right)+O\left(n\right)$
	$T\left(n\right)=t\left(\frac{n}{2}\right)+n+n$
	:
	:
	$\left(n\right)=t(\frac{n}{n})+n+n$
	T(n)=t(1)+n+n
	T(n)=t+n+n
	Where *t* is ignored; therefore, we get
	T(n)=n+n
	$let\;n=k\;\&\;k=\log_{n}$
	$T\left(n\right)=O\left(\log_{n}+n\right)$
